# Novel Immune-Related Gene-Based Signature Characterizing an Inflamed Microenvironment Predicts Prognosis and Radiotherapy Efficacy in Glioblastoma

**DOI:** 10.3389/fgene.2021.736187

**Published:** 2022-01-17

**Authors:** Hang Ji, Hongtao Zhao, Jiaqi Jin, Zhihui Liu, Xin Gao, Fang Wang, Jiawei Dong, Xiuwei Yan, Jiheng Zhang, Nan Wang, Jianyang Du, Shaoshan Hu

**Affiliations:** ^1^ Department of Neurosurgery, Zhejiang Provincial People’s Hospital, Hangzhou, China; ^2^ Department of Neurosurgery, The Second Affiliated Hospital of Harbin Medical University, Harbin, China; ^3^ Translational Medicine Research and Cooperation Center of Northern China, Heilongjiang Academy of Medical Sciences, Harbin, China; ^4^ The Key Laboratory of Myocardial Ischemia, Ministry of Education, Harbin, China; ^5^ Department of Neurosurgery, Shandong Provincial Hospital Affiliated to Shandong First Medical University, Jinan, China

**Keywords:** glioblastoma, immune microenvironment, immune-related gene, EGFR, prognosis, immune checkpoint blockade therapy, radiotherapy

## Abstract

Effective treatment of glioblastoma (GBM) remains an open challenge. Given the critical role of the immune microenvironment in the progression of cancers, we aimed to develop an immune-related gene (IRG) signature for predicting prognosis and improving the current treatment paradigm of GBM. Multi-omics data were collected, and various bioinformatics methods, as well as machine learning algorithms, were employed to construct and validate the IRG-based signature and to explore the characteristics of the immune microenvironment of GBM. A five-gene signature (ARPC1B, FCGR2B, NCF2, PLAUR, and S100A11) was identified based on the expression of IRGs, and an effective prognostic risk model was developed. The IRG-based risk model had superior time-dependent prognostic performance compared to well-studied molecular pathology markers. Besides, we found prominent inflamed features in the microenvironment of the high-risk group, including neutrophil infiltration, immune checkpoint expression, and activation of the adaptive immune response, which may be associated with increased hypoxia, epidermal growth factor receptor (EGFR) wild type, and necrosis. Notably, the IRG-based risk model had the potential to predict the effectiveness of radiotherapy. Together, our study offers insights into the immune microenvironment of GBM and provides useful information for clinical management of this desperate disease.

## Introduction

GBM is the most common and devastating primary brain malignancy in adults, with a median overall survival (OS) of 14.6 months, and only 5.8% of patients survive beyond 5 years despite standardized treatment (Jiang et al., 2016; Siegelin et al., 2021). Therapeutic regimens for progressive or recurrent GBM are even limited, which virtually occur in most patients (Geraldo et al., 2019; Tan et al., 2020). Currently, there are well-studied biomarkers effectively predicting prognosis and sensitivity to treatment, including isocitrate dehydrogenase (IDH) mutation, co-deletion of chromosome arms 1p and 19q, mutations of alpha thalassemia/mental retardation syndrome X-linked (ATRX), telomerase reverse transcriptase gene mutation, and methylation of O^6^-methylguanine-DNA methyltransferase promoter (Jiang et al., 2016). As effective treatment of GBM remains formidable, the development of novel tumor biomarkers may help improve the efficacy of current treatment modalities.

The role of the immune system in tumorigenesis and evolution is receiving increasing attention (Chen and Mellman, 2013). Unlike the previous dogma that the central nervous system is an immune-privileged region, the role of the immune system in the progression of GBM has also been appreciated (Engelhardt et al., 2017; Quail and Joyce, 2017). Recent studies have systematically revealed immune “afferent” and “efferent” arms in the central nervous system (CNS) as the basis for the emergence of the anti-tumor immune response (Engelhardt et al., 2017; Qi et al., 2020). Besides, a clinical trial has revealed the association of CD8 T-cell infiltration with improved prognosis in GBM patients, further confirming the survival benefit of an effective anti-tumor immune response (Yang et al., 2010). In response, multiple immune suppressive mechanisms are hijacked by GBM cells for immune evasion (Ahn et al., 2013). However, the failure of interferon-gamma, a potent immune activator, to benefit GBM patients and the association of a fraction of gene markers that efficiently characterize the immune-mediated tumor elimination with poor prognosis in GBM patients are highly suggestive of the uniqueness of the immunological property of GBM (Wolff et al., 2006; Qian et al., 2018; Wang et al., 2019). Nowadays, several immunotherapies, including immune checkpoint blockade (ICB) therapy, have dramatically extended the survival of a fraction of cancer sufferers (Pardoll, 2012; Borghaei et al., 2015; Larkin et al., 2015), while GBM possesses an incredibly low response rate to ICB (Filley et al., 2017; Zhao et al., 2019). Therefore, a comprehensive exploration of the impact of immune genes on the survival of GBM patients would be crucial for an in-depth understanding of the characteristics of the immune microenvironment of GBM and the development of clinically valuable tumor biomarkers.

In this perspective, we have identified five immune-related genes (IRGs) and developed a risk model of valid prognostic value. Our IRG-based risk model performed superiorly in time-dependent survival prediction compared to traditional molecular pathology parameters. In addition, we found that the tumor microenvironment in the high-risk group was characterized by neutrophil infiltration, increased expression of immune checkpoints, and activation of the adaptive immune response and that these inflamed phenotypes may be implicated in intra-tumor hypoxia, vasculature disruption, and necrosis. Moreover, the IRG-based risk model had the potential to determine the effectiveness of radiotherapy. In a word, this work offers insights into the immune microenvironment of GBM and provides potentially valuable information for the treatment of this desperate disease.

## Materials and Methods

### Data Collection and Preprocessing

The bulk mRNA sequencing data of GBM with corresponding demographics were included. Of these, 166 cases retrieved from The Cancer Genome Atlas (TCGA) database (https://www.cancer.gov/) were used as the training dataset, and the remaining cases retrieved from the Chinese Glioma Genome Atlas (CGGA) database (mRNA-seq 325, mRNA-seq 693, and microarray 301, http://www.cgga.org.cn/) were used as the validation dataset. The mRNA-seq data were TPM normalized, and the microarray dataset was log-transformed. A total of 2,498 IRGs were retrieved from the ImmPort Portal (https://www.immport.org/). The somatic mutation profiles and copy number variation (CNV) files were retrieved from the TCGA database.

### Construction and Validation of the IRG-Based Risk Model

An unsupervised class discovery technique was employed to find stable clusters based on the top 300 IRGs ranked by their mean absolute deviation (MAD) values using the R package “ConsensusClusterPlus” (Supplementary Material S1) (Wilkerson and Hayes, 2010). The clustering method was set as “km” (k-means), and the maximum evaluated K was 6. The optimal number of clusters was determined using the “proportion of ambiguous clustering (PAC)” method.

The weighted gene coexpression network analysis (WGCNA) was performed to identify genes characterizing an active immune response of GBM based on the R package “WGCNA” (Langfelder and Horvath, 2008). Module Eigenges (ME) was defined as the first principal component of each gene module and served as the representative for all genes in each module. Gene significance (GS) represents the degree of linear correlation between module gene expression and sample features. Genes with weighted correlation coefficients over 0.8 were extracted for further analysis.

The univariate and multivariate Cox regression analyses were performed sequentially to identify genes with prognostic significance as well as to determine their regression coefficients. Genes with *p*-values <0.05 in the univariate regression analysis were selected for multivariate regression analysis. The coefficients obtained from the regression analysis were used to yield the following risk score equation:

$$\mathrm{risk score}=\sum_{k=1}^{n}\mathrm{regression coefficient}\times \mathrm{gene expression value}$$

Samples were split into the high- and low-risk groups by the median value of the risk score. The Kaplan–Meier (K-M) plots were generated based on the R package “survival”, and the independent prognostic value of the risk score was evaluated by univariate Cox regression analysis. The time-dependent predictive power of the risk model was evaluated using the receiver operative curve (ROC) and the corresponding area under the curve (AUC). The independent prognostic value of the risk group, as well as clinicopathological parameters, was evaluated using the univariate Cox regression analysis.

### Exploration of the Characteristics in the Tumor Immune Microenvironment

The differential gene expression profile was conducted between subgroups using the R packages “limma” and “edgeR” (Robinson et al., 2010; Ritchie et al., 2015). The absolute value of logFC >1.0 and adjust *p*-value <0.05 were set as the cutoff. Functional enrichment analysis was performed based on the R package “clusterProfiler” and the online tool The Database for Annotation, Visualization and Integrated Discovery (DAVID, version 6.8, https://david.ncifcrf.gov/) (Huang et al., 2009a; Huang et al., 2009b). The Gene Ontology (GO) terms including biological process (BP), cell component (CC), molecular function (MF), and Kyoto Encyclopedia of Genes and Genomes (KEGG) and Biocarta pathways were involved. The false discovery rate (FDR) < 0.05 was set as the cutoff. Gene Set Enrichment Analysis (GSEA) was employed to identify differentially activated signaling pathways (Subramanian et al., 2005). Gene sets were permuted 1,000 times. Normalized enrichment scores (NES) were calculated, and FDR < 0.05 was set as the cutoff. To explore immunological characteristics, 103 gene sets involved in the inflammation/innate immune response, antigen presentation, CD8 T cell function, and cytotoxicity were retrieved from the Molecular Signatures Database (MSigDB, v7.4, http://software. broadinstitute.org/gsea/msigdb/index.jsp) (Supplementary Material S1), and the ssGSEA score of the samples was calculated to assess the activation of specific signaling pathways (Liberzon et al., 2011; Hänzelmann et al., 2013). The fraction of immune cells was estimated using the CIBERSORT algorithm (Newman et al., 2015). Samples with *p*-values over 0.05 were filtered. Tracking tumor immunophenotype (TIP) is a system to quantify the activity of each step of the anti-tumor immune response (Xu et al., 2018). The anti-tumor immune response was conceptualized as seven stepwise events including 1) release of cancer cell antigens, 2) cancer antigen presentation, 3) priming and activation, 4) trafficking of immune cells to tumor, 5) infiltration of immune cells into tumor, 6) recognition of cancer cell by T cells, and 7) killing of cancer cells. The TIP score of each stepwise event was evaluated using the R script of TIP.

### The Tumor Genomic Alterations

Somatic mutation data were analyzed using the R package “maftools” (Mayakonda et al., 2018). The differentially mutated genes were calculated using the functions “mafComapre.” The tumor mutational burden (TMB) was defined as the total number of somatic mutations including common substitutions, insertions, and deletions per megabase, and its calculation has been described before (Chalmers et al., 2017). Significant amplifications and deletions of somatic copy numbers were detected using GISTIC 2.0 (Mermel et al., 2011). FDR-q value ≤ 0.05 was set as the cutoff.

### Prediction of ICB Responsiveness

The tumor responsiveness to the ICB therapy was assessed using the machine learning algorithm TIDE, which is a computational method to model the induction of T cell dysfunction and the prevention of T cell infiltration into tumor through analyzing the expression of specific gene signatures in the tumor expression profile (Jiang et al., 2018). Further, the predicted tumor response to ICB was evaluated by an unsupervised subclass mapping method that is designed to determine correspondence or commonality of subtypes found in multiple independent datasets generated on different platforms (Hoshida et al., 2007).

### Statistics

All statistical analysis was conducted using R software (version 4.0.2). The differences in gene expression, ssGSEA score, TIP score, and the fraction of immune infiltration were assessed using a two-tailed Wilcoxon's test. Univariate and multivariate Cox regression analyses were conducted to establish Cox proportional hazard models. Differences in clinicopathological parameters between the high- and low-risk groups were measured using the Wilcoxon test and Fisher's exact test. The K-M analysis and log-rank analyses were employed to evaluate differences in OS. The independent prognostic value of the risk score was assessed using univariate Cox analysis. Pearson correlation analyses were conducted to estimate the correlation between the risk score and the fraction of immune infiltration. The association between epidermal growth factor receptor (EGFR) mutations and immune infiltration was assessed using logistic regression analysis. *p* < 0.05 was considered statistically significant. We marked *p* < 0.05 as *, *p* < 0.01 as **, and *p* < 0.005 as ***.

## Results

### The IRG-Based GBM Stratification

**FIGURE 1:**
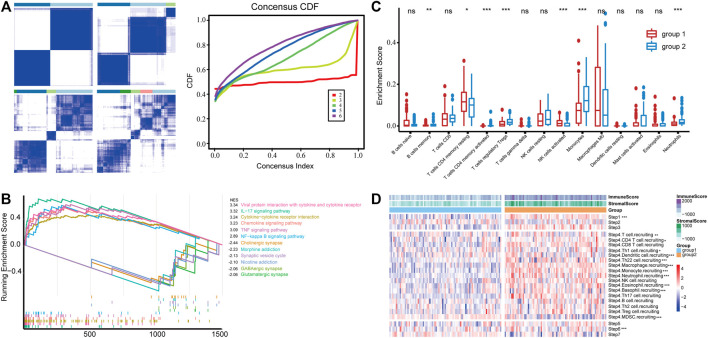
**(A)** Consensus clustering of the GBM samples based on the expression of IRGs, and empirical cumulative distribution function (CDF) corresponding to the entries of consensus matrices. **(B)** GSEA analysis of top pathways enriched in group 1 and group 2, respectively. **(C)** Immune infiltration is estimated by CIBERSORT. Cell with an estimated fraction of 0 in more than half of the samples was filtered. **(D)** The anti-tumor immune response was divided into seven stepwise events, and the activity of each was assessed using the TIP system. *** in Step 6 suggests that group 1 has a significantly higher TIP score than group 2, and the rest indicate higher in group 2.

To obtain GBM subgroups with potential distinct immunological properties, samples were stratified according to the expression of IRGs. The MAD value of 1,288 IRGs with an intersection with the TCGA profile was calculated; thereafter, 166 samples with survival information were stratified based on the expression of top 300 IRGs in terms of MAD value, and two subgroups [termed group 1 (*n* = 87) and group 2 (*n* = 79)] were identified (Figure 1A). Then, we calculated the differentially expressed genes (DEGs, group 2 vs. group 1), performed functional enrichment analysis, and identified inflammatory response, immune response, innate immune response, and neutrophil chemotaxis as the primarily enriched BP, and receptor activity, cytokine activity, and chemokine activity as the main MF (Supplementary Figure S1) implying a difference in immune response between the two groups. Consistently, GSEA analysis revealed that KEGG signaling pathways involved in immune response such as viral protein interaction with cytokine receptor, IL-17 signaling pathway, cytokine–cytokine receptor interaction, chemokine signaling pathway, and tumor necrosis factor (TNF) signaling pathway were significantly enriched in group 2 (Figure 1B), indicating a potentially inflamed microenvironment. Besides, the fraction of immune infiltration was calculated; group 2 was characterized by increased infiltration of B cell memory, T cell CD4 memory activated, Treg, monocyte, and neutrophil, while group 1 had increased infiltration of T cell CD4 memory resting and natural killer (NK) cell (Figure 1C). In particular, quantitative analysis of the activity of the anti-tumor immune response using TIP found that group 2 had increased tumor antigen releasing (step1) and immune cell recruitment activity (step 4) (Figure 1D) but not the infiltration of the immune cell into the tumor (step 5), tumor cell recognition (step 6), and elimination (step 7). In this way, the IRG-based classification yielded two subgroups of GBM, one of which (group 2) contained more inflammatory features in the microenvironment including the activation of immune-related signaling pathways and increased infiltration of neutrophils.

### Construction of the IRG-Based Risk Model

To identify signature genes characterizing such immune-inflamed microenvironment with prognostic significance, the gene expression profile of GBM was divided into 11 modules with functional relevance by WGCNA. Module “magenta” was significantly differentiated by subgroups and highly correlated with group 2 (cor = 0.65) (Supplementary Figures S2A, B). Functional enrichment analysis of the 608 genes included in “magenta” showed that they were mainly involved in leukocyte activation and immune activation (BP) as well as signaling pathways associated with immune cell activation and differentiation (biocarta) (Supplementary Figure S2D). Next, 409 DEGs upregulated in group 2 and the 161 genes that had a weighted correlation coefficient over 0.8 with group 2 were intersected, and 126 genes were obtained as candidate genes for the construction of the risk model (Supplementary Figure S2C). The univariate and multivariate Cox regression analysis identified S100A11, PLAUR, NCF2, FCGR2B, and ARPC1B to be prognostically significant (Table 1).

**TABLE 1:** Identification of signature genes and their regression coefficients.

Gene symbol	Hazard.Ratio.x	CI95.x	P.value.x	Hazard.Ratio.y	CI95.y	P.value.y	Regression coefficient
ARPC1B	1.37	1.1–1.72	0.006	3.05	1.31–7.11	0.01	0.0184
FCGR2B	1.2	1.05–1.37	0.008	1.56	1.02–2.37	0.04	0.0491
NCF2	1.28	1.04–1.57	0.018	3.02	1.3–7.01	0.01	−0.0030
PLAUR	1.44	1.19–1.73	0	2.96	1.44–6.09	0.003	0.4339
S100A11	1.23	1.01–1.51	0.041	0.41	0.22–0.76	0.005	−0.1814

### Increased Risk Score Predicts Poor Outcome

**FIGURE 2:**
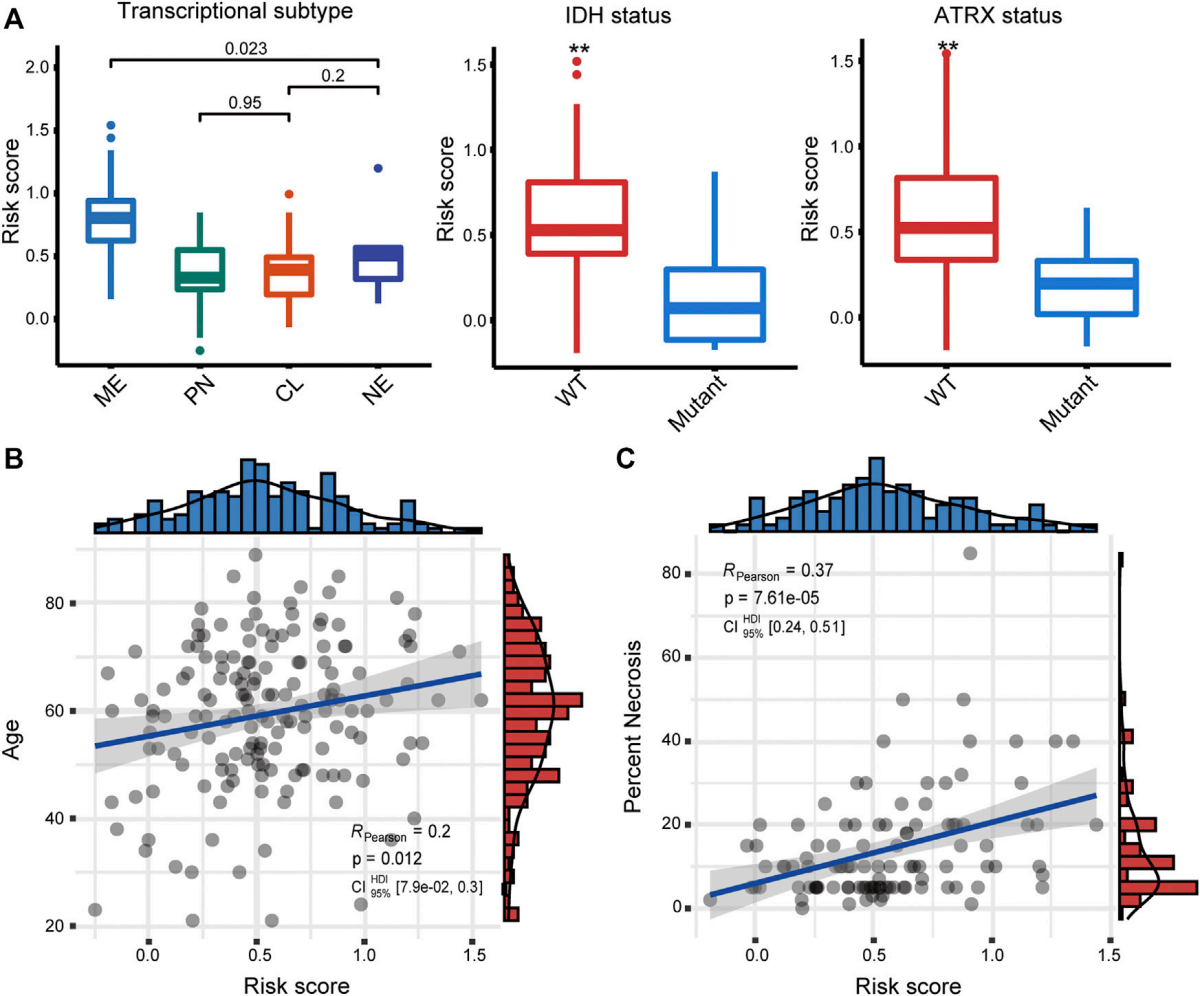
**(A)** Association of the risk score with transcriptome subtype, IDH mutation, and ATRX mutation status. Correlation between the risk score and **(B)** age and **(C)** necrosis. ME, mesenchymal; PN, proneural; CL, classical; NE, neural.

We first examined the relationship between the risk score and the well-studied clinicopathological and molecular parameters of GBM. We found that the mesenchymal subtype had a significantly increased risk score (*p* < 0.001) (Figure 2A), which has the poorest prognosis among the four transcriptional subtypes (Ceccarelli et al., 2016). Besides, the risk score was significantly increased in both IDH wild-type and ATRX wild-type tumors (*p* < 0.01), corroborating the association between the risk score and poor prognosis. Moreover, the risk score was moderately and significantly correlated with age and necrosis percent of the tissue slide (Figures 2B, C), two other factors associated with poor prognosis in GBM patients (Tan et al., 2020).

**FIGURE 3:**
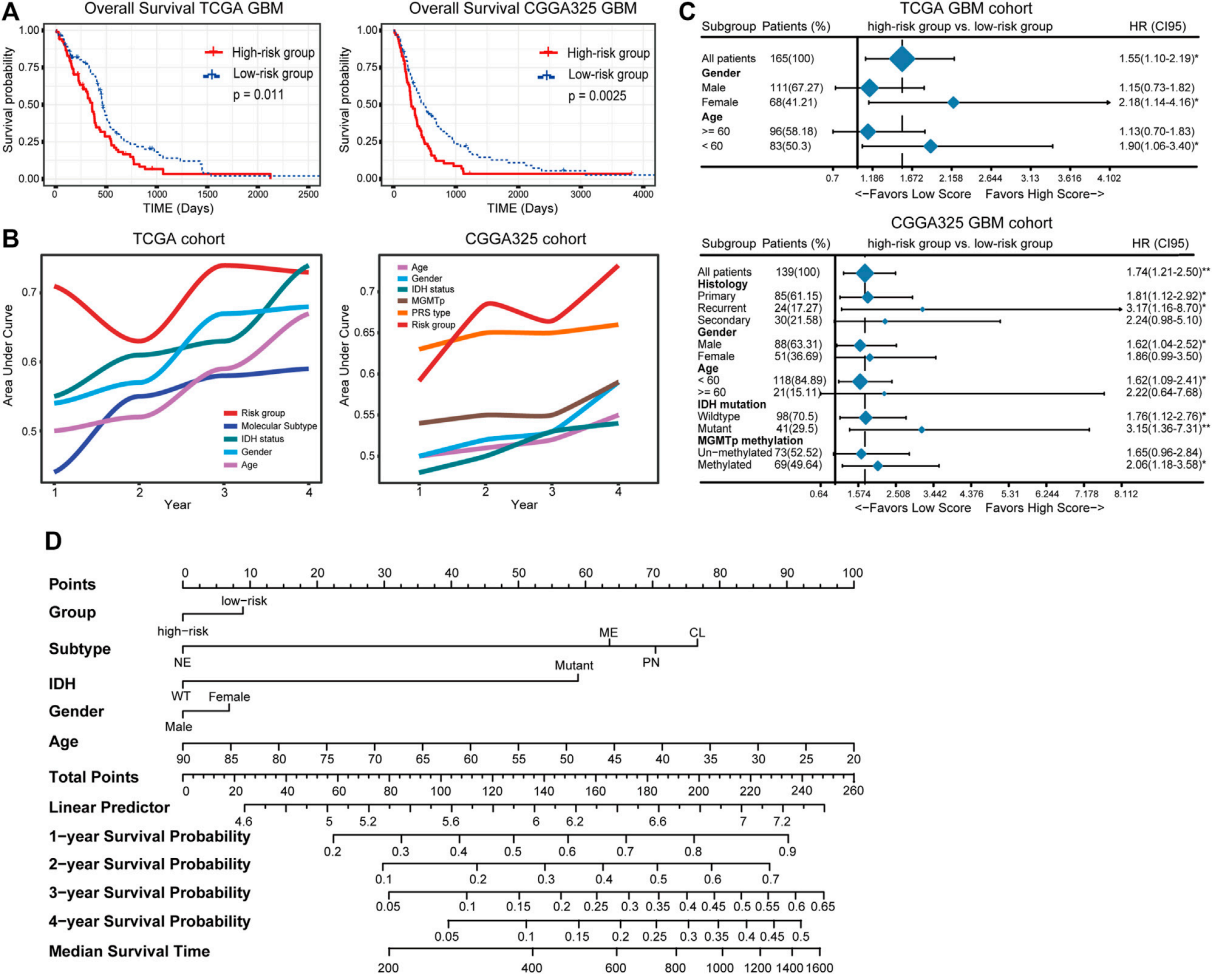
**(A)** The distinct overall survival between the high- and low-risk groups. **(B)** The time-dependent predictive power of the risk model and other prevalent clinicopathological parameters. PRS type: primary, recurrent, secondary type of the tumor. **(C)** The independent prognostic value of the risk score. **(D)** Nomogram demonstrating time-dependent survival rate for patients with different pathological parameters in clinical practice.

Thereafter, samples were split into the high- and low-risk groups based on the median of the risk score (Table 2). The distribution and status of OS showed that GBM patients with increased risk scores had unfavorable prognoses (Supplementary Figure S3A). Likewise, the K-M plots showed that high-risk scores predicted a significantly reduced OS in both cohorts (*p* < 0.05 in the TCGA cohort, *p* < 0.01 in the CGGA325 cohort) (Figure 3A), as well as in two other independent cohorts (Supplementary Figure S3B). The time-dependent predictive power of the risk model was also assessed. The 1-, 2-, 3-, and 4-year AUC values of the risk stratification were 71.24, 63.16, 73.59, and 73.07 in the TCGA cohort, and 59.15, 68.45, 66.45, and 73.20 in the CGGA cohort, performing better than other biomarkers including molecular subtype, IDH mutation status, gender, and age (Figure 3B). Further, the independent prognostic value of the risk score for different populations was determined. Univariate Cox regression analysis found that a high risk score was indicative of an unfavorable prognosis for the total population (HR = 1.55 and 1.74 in the TCGA and CGGA325 cohort) (Figure 3C). In the TCGA cohort, a high risk score was an independent risk factor for female patients and those aged less than 60 years. In the CGGA325 cohort, a high risk score was an independent prognostic factor for primary and recurrent GBM, male, patients aged less than 60, MGMT promoter methylated patients. Nomogram clearly showed that the high-risk group suggests reduced total score and survival (Figure 3D). In sum, these results suggested that the IRG-based risk model had a reliable prognostic value, with high-risk scores reliably indicating a poor prognosis of GBM patients.

**TABLE 2:** Association between the risk group and prevalent clinicopathological parameters.

Term	High-risk group (*n* = 83)	Low-risk group (*n* = 83)	*p*-value
Age	60.54 (±13.23)	58.07 (±13.87)	0.264
Gender			
Male	51 (61.45%)	56 (67.47%)	
Female	32 (38.55%)	27 (32.53%)	0.516
IDH status			
Wild type	77 (92.77%)	71 (85.54%)	
Mutant	2 (2.41%)	9 (10.84%)	0.056
1p19q co-deletion			
co-del	0	0	
Non-codel	81 (97.59%)	79 (95.18%)	NA
MGMTp methylation			
Methylated	22 (26.51%)	33 (39.76%)	
Unmethylated	37 (44.58%)	37 (44.58%)	0.287
TERTp status			
Wild type	1 (1.20%)	4 (4.82%)	
Mutant	16 (19.28%)	16 (19.28%)	0.348
ATRX status			
Wild type	72 (86.75%)	73 (87.95%)	
Mutant	2 (2.41%)	7 (8.43%)	0.17
Tumor purity	0.668 (±0.201)	0.806 (±0.126)	2.14E-06
Subtype			
CL	11 (13.25%)	40 (48.19%)	
ME	59 (71.08%)	14 (16.87%)	
NE	3 (3.61%)	4 (4.82%)	
PN	6 (7.23%)	13 (15.66%)	2.81E-11

### Immunological Characteristics Associated With the IRG-Based Risk Model

**FIGURE 4:**
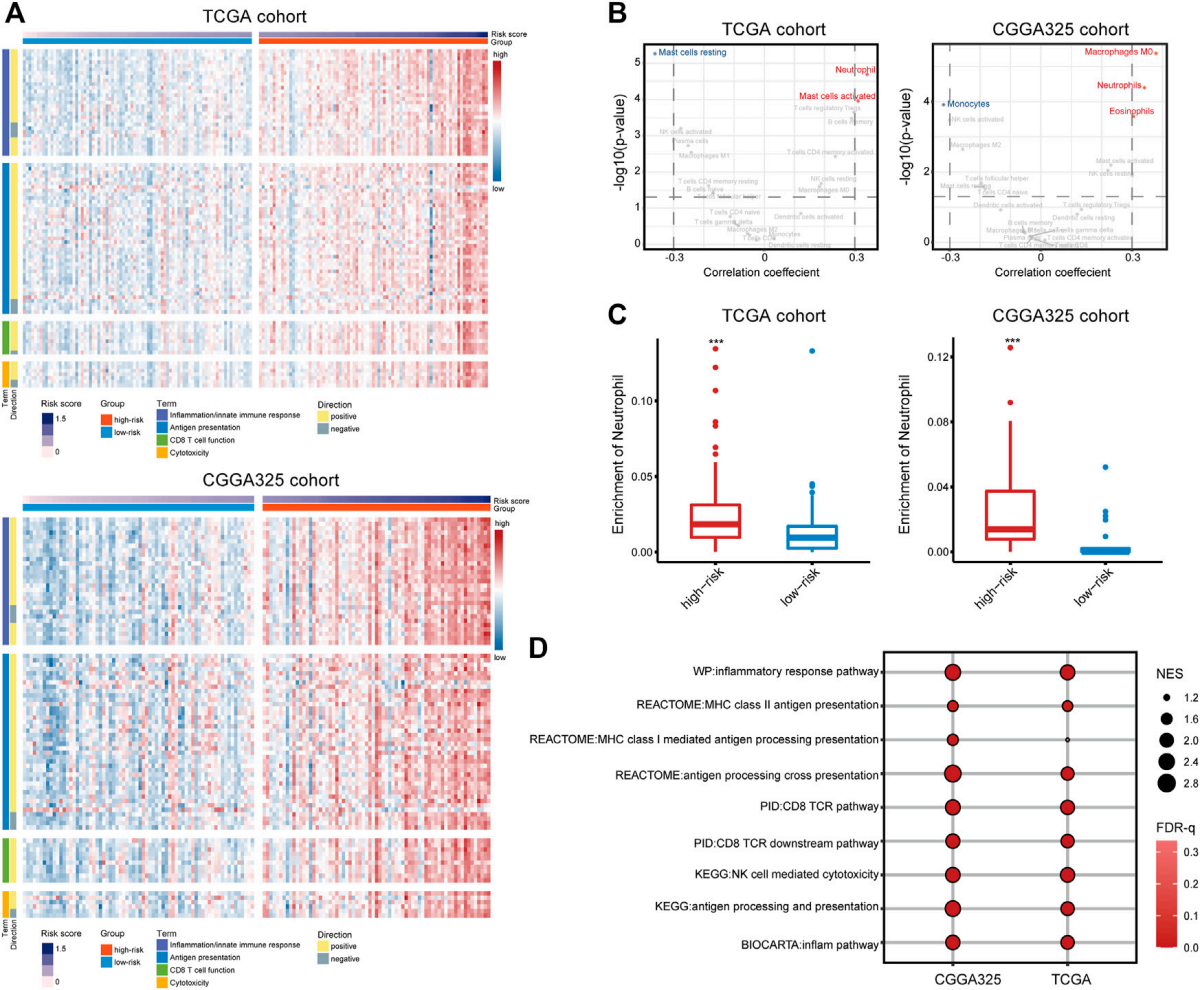
**(A)** The ssGSEA scores of 103 signaling pathways involved in the inflammation/innate immune response, antigen presentation, CD8 T cell activation, and cytotoxicity. **(B)** Correlation analysis of the 22 immune cells estimated by CIBEROST with the risk score. **(C)** The fraction of neutrophils in the high- and low-risk groups. **(D)** GSEA analysis of pathways involved in the anti-tumor immune response.

To investigate the immunological differences between the high- and low-risk groups, the activation of immune-related signaling pathways was assessed. One hundred three gene sets involved in the inflammation and innate immune response, antigen presentation, CD 8 T cell activation, and cytotoxicity were retrieved from the MSigDB database. The ssGSEA score of these signaling pathways increased with the risk score, indicating the immune-activated status of the high-risk group (Figure 4A). Given that immune cells are key players in the immune response (Newman et al., 2015), the immune infiltration fraction was assessed. Consequently, neutrophil was positively correlated with the risk score in both cohorts (cor > 0.3) and significantly enriched in the high-risk group (*p* < 0.001) (Figures 4B, C), suggesting an inflamed microenvironment of the high-risk group. Besides, GSEA analysis found the enrichment of pathways including inflammatory responses, antigen presentation, CD8 T cell activation, and NK cell-mediated cytotoxicity in the high-risk group (Figure 4D), indicating the activation of the adaptive immune response. Further, there are well-established mRNA metrics reflecting the T-cell inflamed immune microenvironment and the cytolytic activity of CD8 T cell and NK cell (Rooney et al., 2015; Danaher et al., 2018). Correlation analysis found a moderate and significant correlation between the risk score and the two metrics (Supplementary Figure S4), corroborating the emergence of the adaptive immune response. Therefore, a high risk score was suggestive of an inflamed tumor microenvironment and the activation of the adaptive immune response.

### Genomic Alterations Associated With the IRG-Based Risk Stratification

**FIGURE 5:**
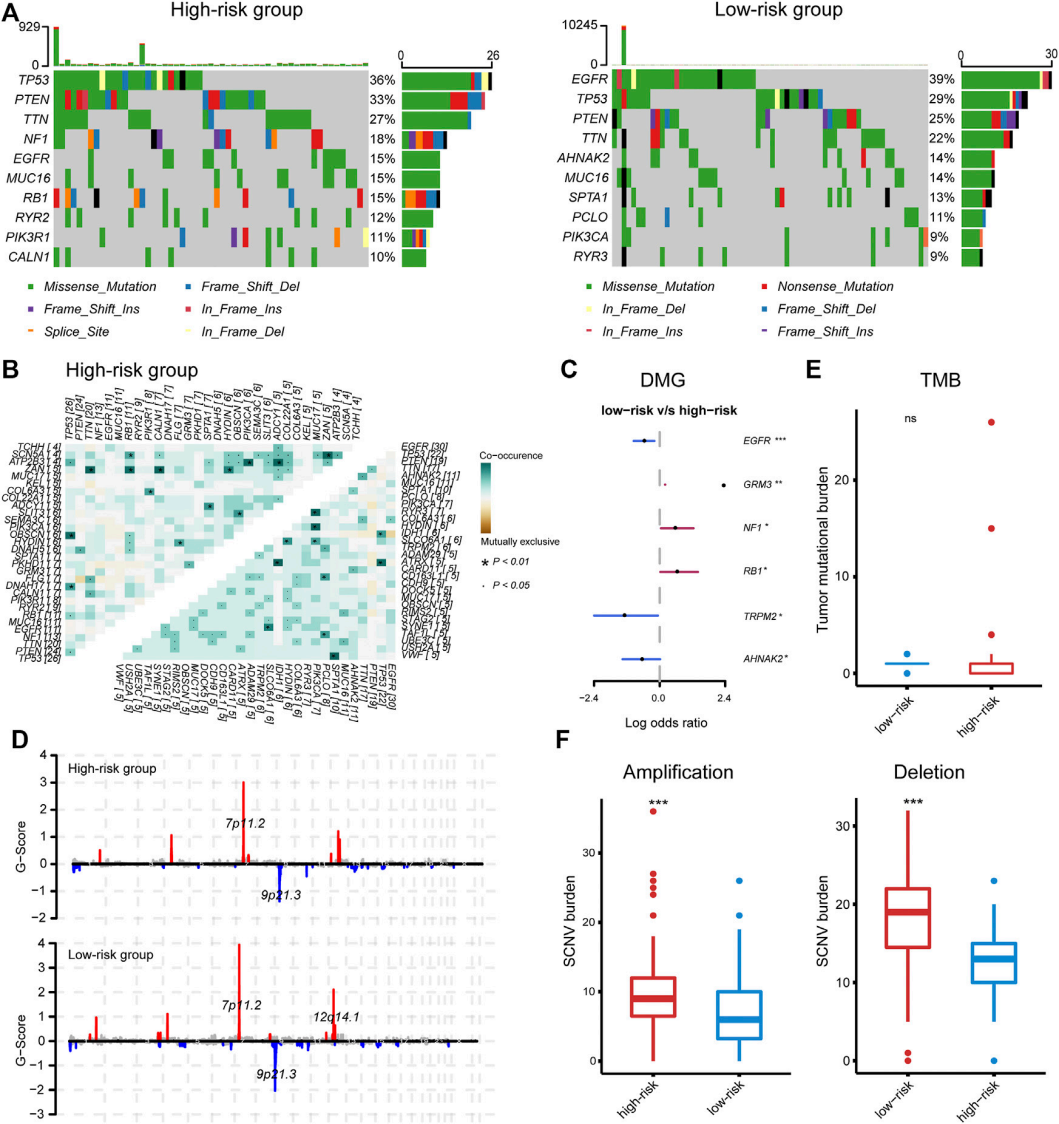
**(A)** The top mutated gene in the high- and low-risk group, respectively. **(B)** Co-occurring and mutually exclusive gene pairs. **(C)** Significantly differentially mutated genes. **(D)** Significant amplifications and deletions in tumor chromosome. **(E, F)** The tumor mutation burden and somatic number alteration load between the high- and low-risk groups.

Given the profound impact of tumor genomic variation on the tumor-immune interaction, we explored the genomic alterations associated with the IRG-based risk stratification. PTEN, TP53, TTN, EGFR, and MUC16 were the top five mutated genes, with a predominance of a missense mutation (Supplementary Figure S5A). We noted some mutations that have been reported to affect immune–tumor interactions; for instance, the mutation frequency of EGFR ranged from 15% in the high-risk group to 39% in the low-risk group, while the frequency of mutations in TP53 and PTEN ranged from 36% to 33% in the high-risk group to 29% and 25% in the low-risk group (Figure 5A). Fisher's exact test found a significantly increased mutation of GRM3, NF1, and RB1 in the high-risk group, and EGFR, TRPM2, and AHNAK2 in the low-risk group (Figure 5C). Among these, EGFR mutation was associated with increased plasma cell infiltration (two-tail Wilcoxon test, *p* = 0.05) (Supplementary Figure S5B). Besides, investigating co-occurrence and mutually exclusive mutations found EGFR and COL6A3 as a co-occurrence gene pair in the low-risk group (Figure 5B), the latter is involved in the production of type IV collagen and is associated with the strength of the vessel wall (Wu et al., 1996). Given the association of EGFR wild type with disrupted vasculature and inflamed microenvironment (Segura-Collar et al., 2021), and the relatively hypoxic microenvironment and hyper-angiogenesis activity of the high-risk group (Supplementary Figure S5D), the inflamed phenotype of the microenvironment in the high-risk group may be associated with EGFR wild-type-mediated aberrant angiogenesis and hypoxia, which partially explained the correlation between the risk score and necrosis.

In terms of CNV, the high- and low-risk groups shared recurring CNVs including 7p11.2 amplification and 9p21.3 deletion, whereas the low-risk group contained an additional significant focal amplification at 12q14.1, leading to the increased expression of CDK4, TSPAN31, MARCH9, and AGAP2 that located at this region (Figure 5D and Supplementary Figure S5C). Despite the individual gene mutations and chromosome alterations, the statistical indicators that related to immune response were also explored. The high-risk group had significantly increased somatic copy number amplification and deletion burden, while the TMB was comparable between the two groups (Figures 5E, F), consistent with our speculation that necrosis may be the source of the activated immune response in the high-risk group.

### Correlation of IRG-Based Risk Stratification With the Efficacy of ICB, Radiotherapy, and Chemotherapy

**FIGURE 6:**
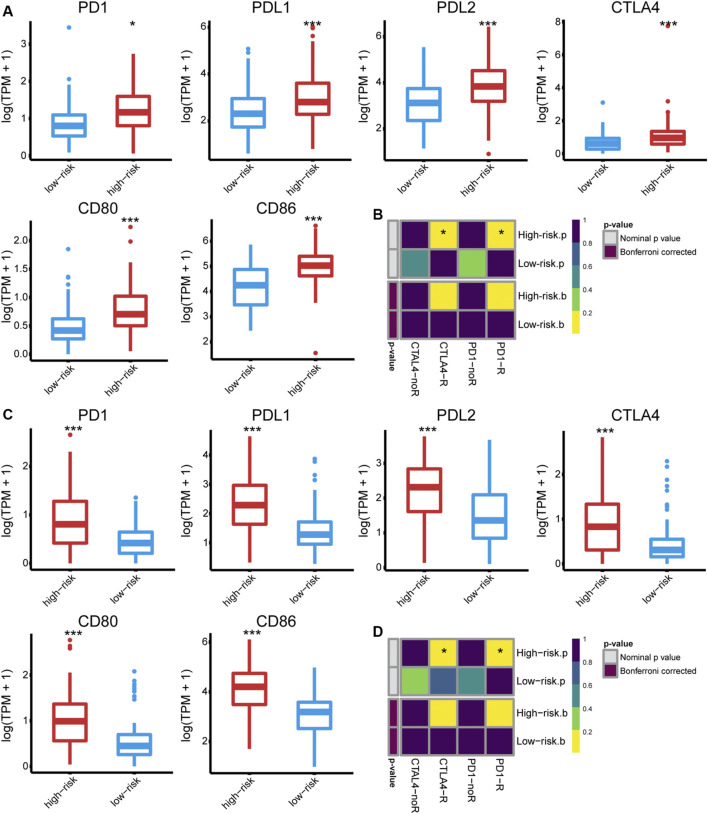
The expression of PD1 and CTLA4, as well as their ligands (PD-L1, PD-L2, CD80, and CD86) in the **(A)** TCGA and **(C)** CGGA325 cohort, respectively. The predicted responsiveness to ICB therapy in the **(B)** TCGA and **(D)** CGGA325 cohort, respectively.

The immune phenotype of tumors is related to their responsiveness to ICB therapy (Chen and Mellman, 2017); we, therefore, assessed the ICB responsiveness of the high- and low-risk groups. The high-risk group had significantly increased expression of PD1 and CTLA4 as well as their ligands (PD-L1/2, CD80/CD86) (Figures 6A, C). Besides, the primary immunodeficiency pathway (KEGG), CTLA4 pathway (Biocarta), and cancer immunotherapy by PD1 blockade (WP) were highly enriched in the high-risk group (Supplementary Figure S6), indicating that immune checkpoints play a role in GBM immune evasion. Tumor response to anti-PD1 and anti-CTLA4 therapies was predicted using TIDE and further validated using SubMap. Consequently, a high risk score failed to predict response to anti-PD1 (Bonferroni corrected *p* = 0.160 and 0.128 in the TCGA and CGGA cohort) or anti-CTLA4 treatment (Bonferroni corrected *p* = 0.208 and 0.120, respectively) (Figures 6B, D).

**FIGURE 7:**
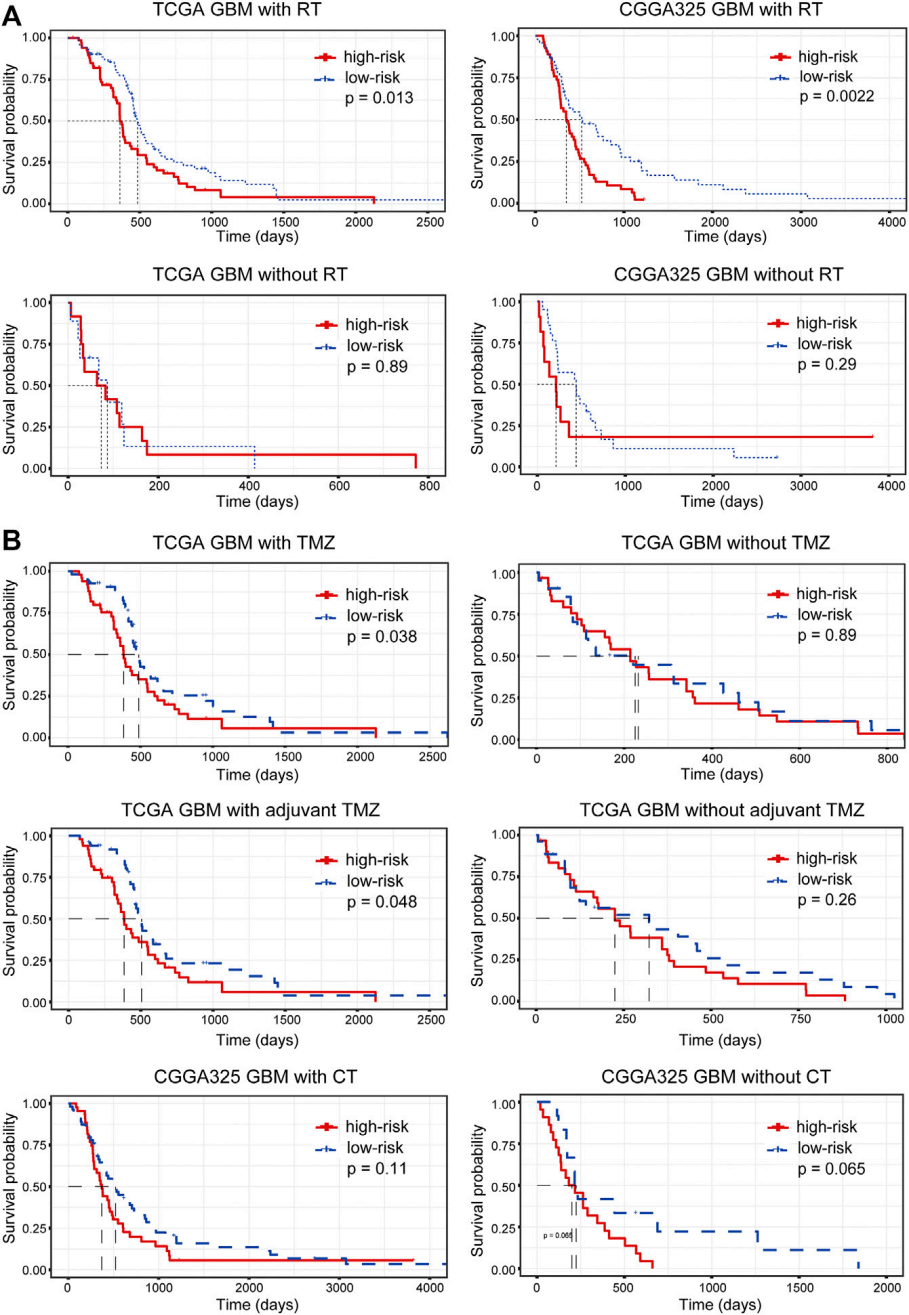
The relationship between risk stratification and the efficacy of **(A)** radiotherapy and **(B)** chemotherapy. RT, radiotherapy; TMZ, temozolomide; CT, chemotherapy.

In addition, we assessed the relationship between the risk stratification and the effectiveness of radiotherapy and chemotherapy. Samples were firstly split into two groups according to their radiotherapy status. For patients receiving radiotherapy, an increased risk score suggested a significantly poor prognosis (*p* = 0.013 and 0.0022 in the TCGA and CGGA cohort, respectively), while the prognostic significance of the risk stratification for patients without radiotherapy was not significant (Figure 7A), indicating that the IRG-based risk model had the potential to predict the efficacy of radiotherapy. Similarly, we grouped samples according to the chemotherapy they received. In the TCGA cohort, lower risk scores for patients treated with temozolomide (TMZ) or adjuvant TMZ predicted increased OS (*p* = 0.038 and 0.048 for patients receiving TMZ and adjuvant TMZ in the TCGA cohort) (Figure 7B). However, the conclusion was not validated in the validation dataset (Figure 7B), suggesting the need to include more samples and to further differentiate chemotherapy modalities to confirm the prediction of chemotherapy efficacy by the risk stratification.

## Discussion

Effective treatment of GBM remains challenging in the modern era. Recently, high-throughput sequencing technology and bioinformatics have helped to identify various biomarkers of clinical significance. Inspired by previous achievements, we have identified a five-gene signature based on the expression of IRGs which showed a solid prognostic value. Besides, we have revealed a relevance between the IRG-based risk stratification and the inflamed tumor microenvironment and that hypoxia, abnormal angiogenesis, and necrosis may be implicated in the activation of the anti-tumor immune response. Despite the fact that our risk model failed to predict the ICB responsiveness and the effectiveness of radiotherapy, it was a potential identifier for the efficacy of radiotherapy. Taken together, our study offers insights into the immune microenvironment of GBM and provides valuable information for improving the current treatment paradigm for this desperate disease.

In this study, we have developed an IRG-based five-gene signature for GBM including ARPC1B, FCGR2B, NCF2, PLAUR, and S100A11 that are involved in the immune response. ARPC1B encodes a subunit of the human Arp2/3 complex involved in the dynamic of the cytoskeleton and the deficiency or loss of which is associated with immunodeficiency (Randzavola et al., 2019; Papadatou et al., 2021). Besides, the mutation of ARPC1B is involved in a novel syndrome characterized by immunodeficiency and spontaneous inflammation that may be attributed to Treg and NK cell dysfunction (Volpi et al., 2019). FCGR2B encodes a receptor for the immunoglobulin gamma complex and regulates the phagocytosis and antibody production of B cells (Xiu et al., 2002). Alterations in the FCGR2B gene are associated with diseases such as systemic lupus erythematosus and rheumatoid arthritis, and its upregulation reduces the sensitivity of lymphoma to rituximab (Lee et al., 2015; Meister et al., 2015; Kim et al., 2016). NCF2 encodes a subunit of NADPH oxidase in neutrophils, inhibition of which may suppress glioma progression. The production of reactive oxygen species (ROS) by neutrophils is primarily dependent on NADPH, meaning that abnormalities in NADPH may lead to severe dysregulation of the inflammatory response (Xu et al., 2019a; Zeng et al., 2019). PLAUR is associated with the malignancy and M2 macrophage infiltration of glioma and acts as an unfavorable prognostic predictor, and the association of PLAUR expression with macrophage infiltration is not limited to tumors (Cancello et al., 2011; Zeng et al., 2021). Besides, the dysregulation of PLAUR is involved in the progression of colon cancer and gefitinib resistance of non-small cell lung cancer (Li et al., 2013; Zhou et al., 2018). S100A11 is an oncogene and encodes a protein that participants in the cell cycle and differentiation. The upregulation of S100A11 promotes the progression of GBM in an NF-kappa B-dependent manner (Tu et al., 2019). Also, S100A11 is involved in the development of hepatocellular carcinoma through inciting inflammation (Sobolewski et al., 2020), substantiating its pro-tumoral role.

In addition to predicting prognosis, immune-related biomarkers are also effective indicators of improved responsiveness to immunotherapy through association with immune-related features (Xu et al., 2019b; Huang et al., 2020; She et al., 2020). ICB is devoted to normalizing the anti-tumor immune response by relieving the “redundant” suppression of CD8 T cells by the tumor microenvironment (Pardoll, 2012). Although it can greatly extend the OS of patients, only a small proportion of patients respond to treatment, especially in GBM. Tumors already possessing a T-cell inflamed phenotype may underlie the efficacy of the treatment, and PD-L1 expression, TMB/TNB, microsatellite instability, and interferon-gamma are also associated with the response to ICB. The ideal conditions would perhaps be for activated CD8 T cells to be suppressed primarily by immune checkpoints, while this is almost inconceivable in GBM. The CNS is compatible with a plethora of immunosuppressive mechanisms to avoid damage caused by excessive immune responses, among which IL10, TGF-beta, VEGF, and COX are often hijacked by tumor cells to evade immune attack, leading to the extremely low response rate of GBM to ICB (Ahn et al., 2013).

There are several well-studied biomarkers related to the immune status and immune microenvironment for the prediction of tumor prognosis and responsiveness to immunotherapy. Cytolytic activity (CYT) is an mRNA metric including granzyme A (GZMA) and perforin (PRF1), which are vital in the cytolytic activity of CD8 T cells and NK cells (Rooney et al., 2015). Impressively, CYT was positively correlated with a variety of factors that enhance tumor immunogenicities, such as oncogenic viruses and neoantigens, and its expression suggested an improved prognosis for a variety of tumors but was reversely correlated with OS in glioma patients, possibly due to the immune response-mediated peri-tumoral edema (Rooney et al., 2015; Wang et al., 2019). Likewise, interferon-gamma (IFNG) response genes are another well-studied mRNA metric for evaluating the potential anti-tumor activity (Ayers et al., 2017). It has been shown that the expression of IFNG gene signature (including IDO1, CXCL10, CXCL9, HLA-DRA, STAT1, and IFNG), as well as expanded IFNG gene signature (including 18 genes involved in the IFNG response and major downstream pathways), characterizes the T-cell inflamed phenotype well, acts as an effective indicator for screen potential responders to ICB therapy, and is a marker of improved prognosis for most tumors, except for gliomas (Danaher et al., 2018; Qian et al., 2018). Besides, another four-gene signature associated with immune pathways and the expression of immune checkpoints also predicts a poor prognosis for patients with lower-grade glioma (Xiao et al., 2020). These results may suggest that excessive immune responses do not benefit glioma patients, even if more tumor cells can be eliminated, in line with our IRG-based gene signature to characterize immune activation while being associated with poor prognosis.

GBM is known as a “cold tumor” with less mutational and neoantigen burden and T lymphocyte infiltration. TMB reflects the production of tumor-associated antigens; while we did not observe a significant difference in TMB between the high- and low-risk groups, an increased tumor antigen releasing has been found in the high-risk group by the TIP system. In addition to the inadequate vascular system and blood–brain barrier in GBM, about 15% of the interstitial fluid wrapped in soluble antigens leaks from brain parenchyma into the cerebrospinal fluid, which in turn is drained into the cervical lymph nodes (Engelhardt et al., 2017). Such an immune “afferent” and “efferent” system, although inefficient, also provides the prerequisite for the activation of the antitumor immune response, and thus the infiltration of CD8 T cells is associated with improved prognosis in patients with newly diagnosed GBM (Yang et al., 2010). Alternatively, necrosis may have induced a violent inflammatory response accompanied by a massive release of cellular contents, and mutations in EGFR may lead to defects in pericyte coverage, which in turn exacerbates hypoxia and necrosis and partially explains the benefit of the adaptive immune response; a spring-up of a massive inflammatory response hardly offsets the damage caused by inflammation (Segura-Collar et al., 2021).

In conclusion, we have constructed an immune-related five-gene signature with solid prognostic value, and there was significant immunological heterogeneity between the high- and low-risk groups. To correct for bias, biological experiments, as well as clinical trials, are needed to validate these results.

## Data Availability

The original contributions presented in the study are included in the article/Supplementary Material; further inquiries can be directed to the corresponding authors.
