# Supplementary material for: Novel Immune-Related Gene-Based Signature Characterizing an Inflamed Microenvironment Predicts Prognosis and Radiotherapy Efficacy in Glioblastoma
Source: Front Genet. 2022 Jan 17;12:736187. doi: 10.3389/fgene.2021.736187 (PMC8801921; doi:10.3389/fgene.2021.736187)
Supplement: Supplementary file 2 [file Table1.DOCX]

Supplementary Material

# Supplementary Figures

## Supplementary Figures

##
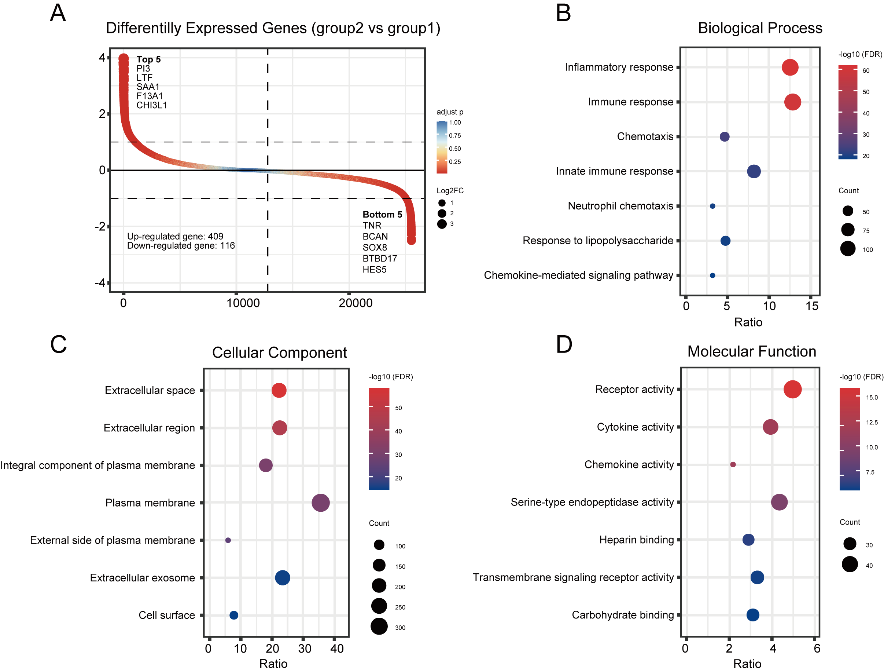


## Supplementary Figure 1. (A) DEGs between the two groups (group 2 vs. group 1). Functional enrichment analysis including (B) BP, (C) CC, and (D) MF.


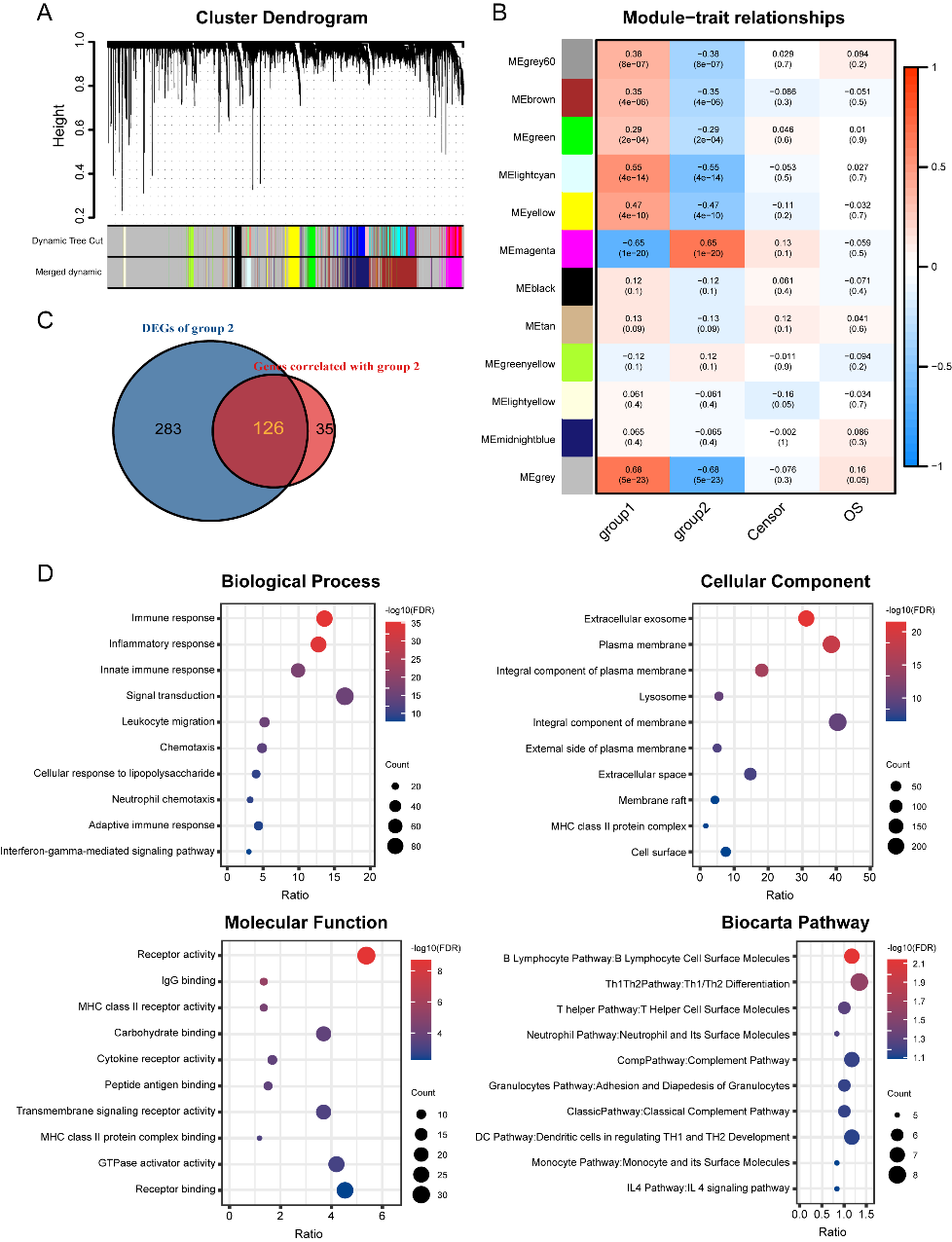


**Supplementary figure 2**. (**A**) The cluster dendrogram. (**B**) The relationship between gene modules with clinical traits including IRG-based subgroups and survival. (**C**) Venn diagram demonstrating the selection of candidate genes for Cox regression analysis. (**D**) Functional enrichment analysis of the genes involved in the module ‘magenta’.


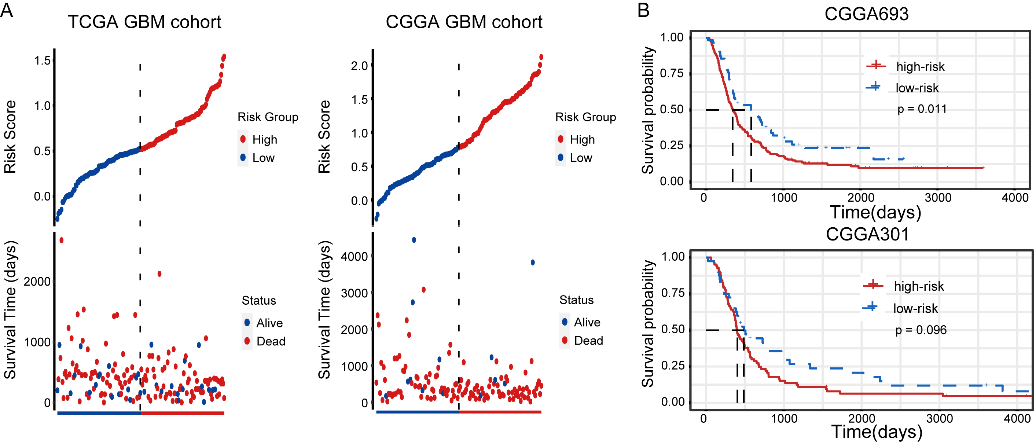


**Supplementary figure 3**. (**A**) The distribution of overall survival and status with increased risk score. (**B**) The prognostic value of the risk model in the other two independent cohorts.


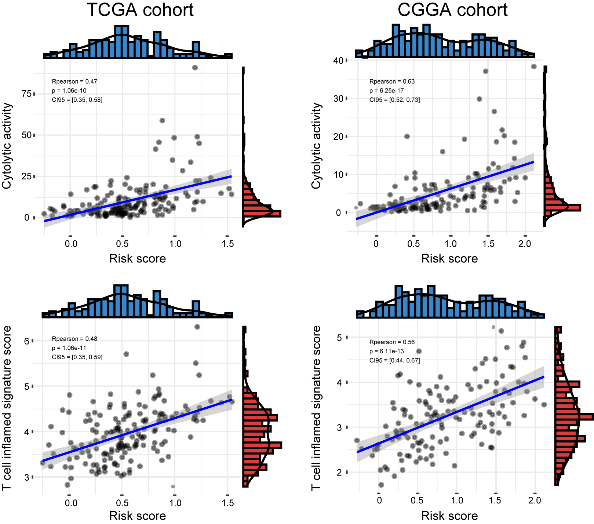


**Supplementary figure 4**. Correlation between the risk score with cytolytic activity and T cell inflamed signature score.


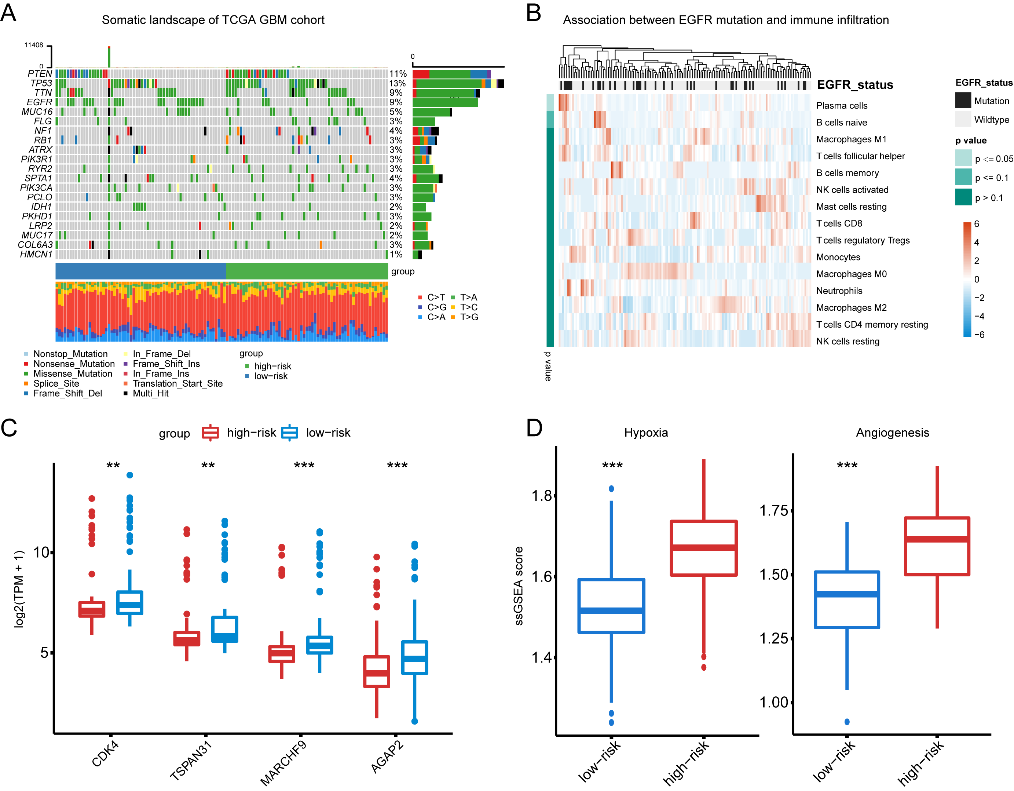


**Supplementary figure 5**. (**A**) The top 20 mutated genes ranked by their mutation frequency. (**B**) The relationship between EGFR mutation and immune infiltration. (**C**) The expression of CDK4, TSPAN31, MARCHF9, and AGAP2 that located at 12q14.1. (**D**) Comparison of the activation of hallmark hypoxia and angiogenesis pathway between the high- and low-risk groups.


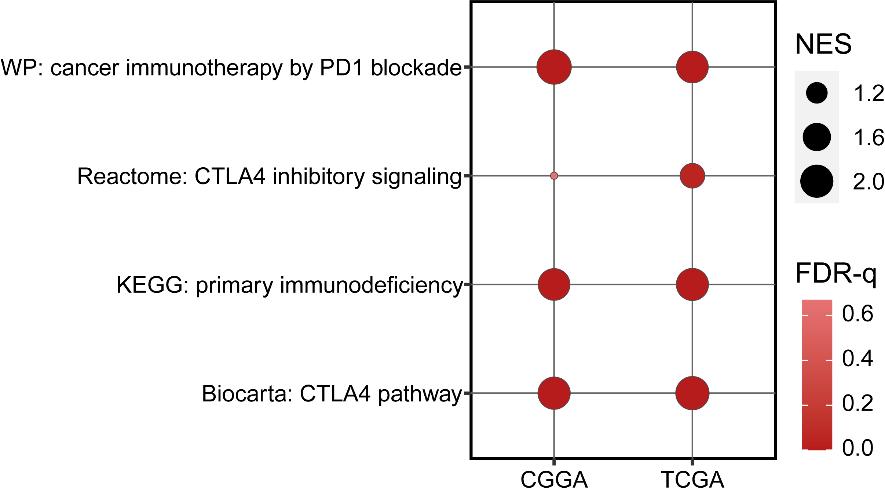


**Supplementary figure 6**. The enrichment of immune checkpoint-mediated pathways in the high-risk group.
